# Understanding UX Better: A New Technique to Go beyond Emotion Assessment

**DOI:** 10.3390/s21217183

**Published:** 2021-10-29

**Authors:** Leonardo Marques, Patrícia Gomes Matsubara, Walter Takashi Nakamura, Bruna Moraes Ferreira, Igor Scaliante Wiese, Bruno Freitas Gadelha, Luciana Martinez Zaina, David Redmiles, Tayana Uchôa Conte

**Affiliations:** 1 Institute of Computing (IComp), Federal University of Amazonas (UFAM), Avenida Rodrigo Otávio 6200, Manaus 69067-005, Brazil; patriciagmf@icomp.ufam.edu.br (P.G.M.); walter@icomp.ufam.edu.br (W.T.N.); bruno@icomp.ufam.edu.br (B.F.G.); tayana@icomp.ufam.edu.br (T.U.C.); 2 Department of Informatics, Pontifical Catholic University of Rio de Janeiro (PUC-Rio), Rua Marquês de São Vicente 225, Rio de Janeiro 22451-900, Brazil; bruna.antonelli2@gmail.com; 3 Academic Department of Computing (DACOM), Federal University of Technology of Paraná (UTFPR), Campo Mourão 87301-899, Brazil; igor.wiese@gmail.com; 4 Departament of Computing, Federal University of São Carlos (UFSCar), Sorocaba 18052-780, Brazil; lzaina@ufscar.br; 5 Department of Informatics, University of California Irvine (UCI), Irvine, CA 92697, USA; redmiles@ics.uci.edu

**Keywords:** user experience, UX, UX technique, UX evaluation, industrial study, comparative study, HCI

## Abstract

User experience (UX) is a quality aspect that considers the emotions evoked by the system, extending the usability concept beyond effectiveness, efficiency, and satisfaction. Practitioners and researchers are aware of the importance of evaluating UX. Thus, UX evaluation is a growing field with diverse approaches. Despite various approaches, most of them produce a general indication of the experience as a result and do not seek to capture the problem that gave rise to the bad UX. This information makes it difficult to obtain relevant results to improve the application, making it challenging to identify what caused a negative user experience. To address this gap, we developed a UX evaluation technique called UX-Tips. This paper presents UX-Tips and reports two empirical studies performed in an academic and an industrial setting to evaluate it. Our results show that UX-Tips had good performance in terms of efficiency and effectiveness, making it possible to identify the causes that led to a negative user experience, and it was easy to use. In this sense, we present a new technique suitable for use in both academic and industrial settings, allowing UX evaluation and finding the problems that may lead to a negative experience.

## 1. Introduction

To be successful, interactive systems need to fulfill user expectations and create a positive User eXperience (UX). Thence, UX has attracted increasing interest in recent years [1], extending the perspective on usability to less pragmatic, more hedonic, and non-task-oriented considerations about interactive systems [2,3]. This growth exists both in academia and industry, evidenced by increasing numbers of UX-related degrees at undergraduate and graduate levels and by high job demand [4]. According to Petterson et al. [1], the term “user experience” roughly returns 20,800 results for the publication year 2010, increasing to more than 32,500 for the year 2016 (+56% in total). Despite this, it remains a challenging and actively discussed area for both researchers in academia and practitioners in the industry [1].

The additional focus of UX on hedonic attributes led researchers to develop techniques focusing on these attributes [5]. For instance, the most commonly used dimensions as UX quality indicators are “affect” and “aesthetics” [6]. Additionally, scales are the most frequently used methods to measure those dimensions, to evaluate both specific UX dimensions or UX in general [1]. However, scales may be a relatively simplistic form of measuring emotions [6] and provide narrow data to help understand what may have caused a negative UX [7].

Often, a follow-up interview is needed to provide a better understanding of the research findings obtained through scales [8]. The development of an integrated technique that allows understanding the data without the need to use an additional technique is an important factor to be considered, as it reduces the need to use a complementary approach to understand the results obtained through scale-type techniques (as an interview). An integrated technique can provide several advantages, among which, the decrease in the user’s cognitive load when participating in a UX assessment stands out. In addition, UX is multidisciplinary and can be studied in an intersection of areas, such as cognitive science, design, psychology, and engineering, with different particularities in each area [1].

Thus, defining what to evaluate in a given context is essential when considering UX. In this sense, another problem is that few UX techniques are specifically designed for the evaluation of software applications [9]. Consequently, there is little evidence that the existing techniques enable identifying specific problems that may have affected the UX when interacting with a software application. For instance, a generic UX assessment technique, that is, designed to assess UX in various contexts, does not address issues specific to software applications, such as good battery management (see Section 3).

Concerning the industrial context, the main interest when considering UX is in designing better products [10]. Professionals are looking for a definition that considers various aspects of interaction [10], and methods that are simple and inexpensive to use [11]. Practitioners also prefer to use a qualitative approach, as it provides more relevant information about the application [10,11].

Considering the issues presented above, we focus in this paper on providing an approach called UX-Tips (User eXperience Technique for Interactive ProductS). With UX-Tips, we aim to provide a technique specifically focused on the context of software applications (desktop and mobile). Furthermore, we intend to better understand the results of a UX evaluation by identifying the reasons that caused a negative UX. To do that, UX-Tips considers various aspects of interaction [10] and provides a way to collect qualitative data [11].

We developed the first version of UX-Tips and assessed its feasibility in a study [12]. In addition to verifying the feasibility of UX-Tips, we wanted to gain insights for improvement to develop a final version of the technique that is more robust and cohesive with the gaps identified in the literature, which we present in this article. In this sense, we investigated the following research questions:

- RQ1: Does UX-Tips allow users to report issues that caused a negative UX?
- RQ2: How does UX-Tips meet industry interests when evaluating UX?

To answer these questions, we conducted two empirical studies to verify our technique’s performance in terms of efficiency and effectiveness. These metrics make it possible to investigate whether to evaluate UX without spending much time on the evaluation and find the problems that affected UX. We decided to present the studies separately, as they consider different perspectives (academy and industry) and different categories of users. For the first (Section 4), we compared UX-Tips to another technique named Integrative Heuristic Inspection (IHI) [13] using both of them in a UX evaluation. For the second (Section 5), we tested UX-Tips in an industrial setting to verify its viability in a real development context by evaluating a mobile application under development. In Section 6, we discuss the contributions we identified considering the results obtained in both studies.

The contributions of this article are two-fold: (1) to provide a technique with a theoretical foundation (since it is a gap found in literature [8]) that guides users to report their experiences in detail, indicating what they consider a problem and how they felt about it; and (2) to provide evidence that UX-Tips meets the needs discussed in the literature (e.g., presenting empirical validation, being easy to use, helping to identify problems that may lead to a negative UX, and providing qualitative results).

In addition to using UX-Tips in the academic context, in this article, we present the practical results of its applicability in a real context of use in the software industry. In addition, the contributions of this article are fundamental to show the use of UX-Tips by industry professionals in a real context, analyzing the feedback provided by them on the applicability of the technique in practice.

## 2. Background and Related Work

In the 1990s, the main focus of the Human–Computer Interaction (HCI) community was on usability, whose proposal according to [14] is to: ”estimate the degree to which specific users can use a software product to achieve specific goals effectively, efficiently and satisfactorily in a specific context of use”. Figueroa et al. [15] considered that this definition makes very clear the need to evaluate a software product to establish whether or not it is usable. Despite HCI’s focus on usability, in the 1990s experiential marketing pointed out that customers want products “that dazzle their senses, touch their hearts and stimulate their minds” [16].

In this sense, HCI became interested in how people “feel” while and as a consequence of engaging with technology [17]. Taking into account that usability was too narrow to represent the experience of users, Don Norman argued about the need for introducing a new term that encompasses all the aspects of user interactions [18]. As stated by Hassenzahl [19], a product should no longer be seen as simply delivering a bundle of functional features and benefits—it provides experiences. In this context, usability is one aspect of UX among others, such as interaction design and user research, i.e., UX includes usability [20]. Furthermore, one of the core pillars of academic UX research is UX evaluation [1,21].

In the literature, the most accepted UX construct was defined by Hassenzahl [19], where he defines that a product provides a good UX when it obtains good indicators in two significant aspects, which he calls pragmatic and hedonic aspects. Hasssenzahl [19] stated that a product might be perceived as pragmatic because it provides effective and efficient means to manipulate the environment. On the other hand, a product may be perceived as hedonic because it provides stimulation, identification, or provokes memories. For instance, ease of learning and efficiency are criteria related to the pragmatic aspects, while aesthetics, novelty, and attractiveness are criteria related to the hedonic aspects.

### 2.1. UX Research

Since its idealization, some of the key challenges faced by UX research remain open. For instance, Bargas-Avila and Hornbæk [6] indicated that, although many papers discuss UX, its definition and distinct characteristics as a research field are currently unclear, and these difficulties remain a challenge for UX research [1,6,22]. Many methods have been proposed. However, as Roto et al. [23] indicated, none of them have general acceptance. This lack of consensus led to methods with a high degree of variety, which is noticeable in studies to categorize them [1,6,8].

Some of the studies and categorizations have limitations. For instance, in the Bargas-Avila and Hornbæk [6] mentioned above, they identified three limitations in methodological approaches. First, the proposals of new methodologies are seldom validated. In UX, comparisons among methodologies are rare and, when existent, weak. Second, some methods (sketches, probes) also raise discussions about the validity of the interpretation of their results. Finally, some publications argue about the need to rely on methods that allow the users to report their experiences instead of methods that focus on analyzing their behavior.

Vermeeren et al. [8] investigated a total of 96 UX evaluation methods used in academia and industry, identified through literature reviews, workshops, group sessions, and online searches. They concluded that it is necessary to develop methods with a theoretical foundation, considering different facets of the experience and the context for which the method is developed. In addition, the authors emphasize the need for easy-to-use methods with easy to analyze results.

Petterson et al. [1] analyzed UX evaluation by reviewing 100 papers from 2010 to 2016 and presented four relevant findings. First, there was a growing number of papers over these years. Second, despite the overall increasing trend, only a quarter of the papers referenced the UX literature. This result reiterates the need for more methods with a theoretical foundation, as pointed out by Vermeeren et al. [8]. Third, scales were the most used method in UX evaluation, but their results had to be complemented with interviews to improve the comprehension of the problems. Fourth, the study confirms the need for methods that help to identify both the emotions and the problems that evoked them, reducing the need to use multiple methods.

### 2.2. UX in Practice

Despite the greater interest in UX, only recently has this led to an increasing job demand in the industry with claims that UX design is a strategic advantage in companies [4]. This growth has aroused interest in UX in the industrial context.

Roto et al. [24] investigated 30 UX assessment methods used in both academia and industry. According to them, the main requirement for UX evaluation methods in the industry is that they should be inexpensive, i.e., do not require many resources, are fast, and are simple to use. Qualitative methods are preferred in the early stages of development to provide relevant information about application design [10,11].

Lallemand et al. [10] identified that the industry’s primary interest in UX is to design better products. There is also a concern with the various aspects of interaction. Ardito et al. [11] argued that developers are more aware of the importance of UX in the development process, but few methods are simple and do not require many resources to use.

In summary, we may notice that some gaps, even reported in previous works, persist and are discussed in more recent papers, such as the lack of (a) methods that are empirically validated and allow users to describe their experiences [6]; (b) methods that provide theoretical grounding and are easy to use [8]; (c) methods that help to identify both emotional aspects and application problems in order to avoid the use of multiple methods [1]; and (d) methods that allow collecting qualitative data, which help to understand the results of the UX assessment. In order to mitigate these gaps, we propose UX-Tips, and we present it in the next section.

## 3. Presenting UX-Tips

In this section, we present our proposed technique UX-Tips, which is differentiated from other techniques because it is focused in a specific context, i.e., software applications and considering specific software issues, such as interface, innovation, physical characteristics (in the case of mobile application), learning and ease of use of the application, efficiency of use and data processing, and other more general dimensions of the UX, but with the description of the assessment items focused on software aspects (see Section 3.2 for more details). In addition, its conception is grounded on the UX literature, as we present in the following subsection.

UX-Tips consider various aspects of UX and aiming at enabling the identification of the specific problems that hampered UX. Additionally, due to the subjective and multidisciplinary nature of UX [1], it is necessary to consider the development of methods that allow the UX evaluation from different points of view, from the actors involved in the software development, as well as its end users. UX-Tips benefits from using a mixed-methods approach to address this gap, allowing for assessment through inspection (professional assessment) and testing (user assessment). We present more details about the use of UX-Tips in the Section 3.3, and we use UX-Tips in inspection and test in Section 5. The raw data study can be found in the Supplementary Materials.

### 3.1. UX-Tips Theoretical Foundation

We built the theoretical foundation based on the work of Vermeeren et al. [8], since it is one of the most cited works that investigated and characterized UX methods. As a systematic mapping study, the results collected by Vermeeren et al. [8] encompass a comprehensive set of techniques, which allow for substantial completeness of techniques and, therefore, a significant base.

First, we collected all UX dimensions reported in the publications investigated by Vermeeren et al. [8] to create our theoretical basis. Then, we gathered all dimensions as long as there was a clear definition of their meaning. We did this in the process of dimension inclusion because assessing the dimension definition is an essential aspect. It allows us to know what a dimension aims to assess precisely, which might not be so clear if the dimension was only named.

**Figure 1:**
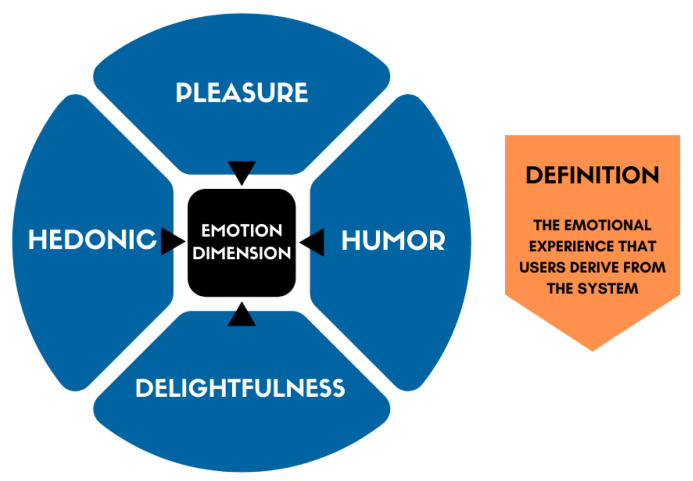
Example of the emotion dimension grouping.

Figure 1 presents an illustrative example of the process we did, instantiating the emotion dimension. After collecting all dimensions, we analyzed and organized them into groups of related dimensions, i.e., we integrated each dimension (in blue—see Figure 1) that evaluates the same aspects of an application (according to their original definitions) into the same group. Consequently, we chose the dimension that presented a better description (in orange) to represent all the dimensions integrated into its group (in black).

After grouping all dimensions, we performed a validation process. This process consisted of a detailed review of each dimension by two UX specialist researchers. Each researcher carried out their review in two separate sessions, where they checked if the grouped dimensions were consistent.

The second part involved the creation of evaluative items, i.e., items that work as heuristics that assess whether an application is adequate to the items or not. We created items based on the definitions of each dimension. In this part, one UX specialist derived the items based on the dimension’s definition. Then, three UX specialists analyzed the description of each item and its agreement with the dimension’s definitions through different meetings until they reached a consensus.

While validating the grouping of dimensions, we found that some aspects were not suitable for the context of our technique (software applications). For example, according to the definition of the emotion dimension (collected in literature), it is necessary to analyze whether the application is funny. However, considering the context for which we developed UX-Tips (evaluating software applications), it is not suitable to assess whether the application is funny or not (as long as being funny is not its purpose). Instead, it is more fitting to assess whether it allows the user to feel happy to use it.

In this sense, we created the item EMT2 since funny is not always applicable. For this reason, we made a recast to fit it into the context of the technique. In the following subsections, we present each dimension we included in the UX-Tips technique, describing their goals. For each dimension presented, a table contains the evaluative items that make up each technique dimension.

We analyzed the suitability to the context for each dimension during the meetings with the specialists (in the validation process). Therefore, the items in the tables will not always syntactically reflect the description of the dimension, but they encompass all the characteristics presented in it.

### 3.2. UX-Tips Dimensions

UX-Tips is composed of 13 UX dimensions with 29 evaluative items. The dimensions are Aesthetics, Emotion, Engagement, Innovative, Social, Physical Characteristics, Utility, Control, Learning and Ease of Use, Efficiency, Feedback, Value-Added, and Satisfaction. In the following subsections, we present the definition of the dimensions that make up the UX-Tips technique and its evaluative items (in tables).

#### 3.2.1. Aesthetics Dimension

The first dimension is Aesthetics, and its goal is to evaluate specific characteristics of the interface, such as the screen layout (see Table 1). All dimensions that make up the Aesthetics dimension have, in their definitions, characteristics that evaluate aspects related to the visual appearance and aesthetics of the interface [25]. According to the definitions of all dimensions integrated into Aesthetics, an application provides a good experience when its interface is organized and clear. Evaluating the aesthetics of the application can be essential to check whether the interface pleased the user or not. Table 1 shows the Aesthetics’ dimension items.

#### 3.2.2. Emotion Dimension

The Emotion dimension is related to the user’s emotional experience as a consequence of the interactionVatanasuk et al. [26]. Emotions can be both positive and negative. Any emotional characteristic evoked in the user by the system is considered an emotion. Some emotions identified in the definitions of the collected dimensions involve verifying if the product is fun, charming, and meets user expectations. In addition, factors include checking for negative emotions, such as frustration, disappointment, irritation, and discouragement. Table 2 shows the Emotion’ dimension items.

#### 3.2.3. Engagement Dimension

The Engagement dimension represents the aspects that lead the user to come back to the product, i.e., if the product has awakened the desire to use it again or even to purchase it [25]. In addition, this dimension also assesses the characteristics that motivate the user to continue using the product for a longer time [27]. Any stimuli aroused by the system in the user, such as curiosity and interest, are evaluated by this dimension. These stimuli can awaken the user’s intention to proceed with the use of the product. Assessing the engaging characteristics of a product can be essential to investigate if it allows the user to have fun. Table 3 shows the Engagement’ dimension items.

#### 3.2.4. Innovative Dimension

The Innovative dimension refers to whether it was creatively designed to the point of getting the user’s attention. If a product has important features that are innovative and allow users to explore something they did not expect, this product can be considered innovative [28]. Table 4 shows the Innovative’ dimension items.

#### 3.2.5. Social Dimension

The Social dimension is related to the user’s interaction with other people through the product. The definitions of the dimensions we collected and grouped in Social indicated that it is relevant to evaluate whether the product allows users to share their experiences with others [29]. Additionally, this includes the assessment of whether the product is used by other people. Finally, this dimension also involves assessing whether the product allows the user to be up-to-date on a hot topic. Table 5 shows the Social’ dimension items.

#### 3.2.6. Physical Characteristics Dimension

The Physical Characteristics dimension is usually related to checking if the weight, size, and dimensions of the product please the user [30]. Physical characteristics may vary depending on the type of product evaluated. A mobile device has different sizes and dimensions compared to a computer. Evaluating these characteristics can be essential to deliver a product that complies with users’ wishes. For example, checking battery life or the size of a keyboard can help make the product more suited to the user’s tastes. Another characteristic observed in this dimension is the efforts or physical activities required from the user when interacting with the product. These characteristics can influence the user’s final decision to use the product or not. Table 6 shows the Physical Characteristics’ dimension items.

#### 3.2.7. Utility Dimension

The Utility dimension helps assess whether the product has proved useful to users, i.e., by allowing the user to perform his tasks or serve a relevant function. Thus, the product becomes useful since it can help in aspects that can be important to users [31]. Table 7 shows the Utility’ dimension items.

#### 3.2.8. Control Dimension

Allowing the user to control their interaction with the product can impact choosing between one product or another. In this sense, the Control dimension assesses whether the user felt in control of the interaction [25]. A flexible product that allows users to choose how to interact is likely to be their option. Therefore, this is a relevant factor when evaluating the quality of a product. Table 8 shows the Control’ dimension items.

#### 3.2.9. Learning and Ease of Use Dimension

The Learning and Ease of Use dimension refer to whether the product allows for an interaction-free of obstacles. In addition, this dimension also assesses the ease of learning to use the product, i.e., if the product provides sufficient conditions to promote learning its features in the first use. In the grouping stage, we noted that some references generally included two characteristics in their definitions. The first one is to assess the ease of use of the product, related to assessing how easy the application presents itself to the user on first use. The second characteristic is to assess the ease of learning to use the product, related to assessing whether the application has features that help learn to use it, such as providing guides. However, there was no reference in which the name expressed the idea of evaluating these two characteristics. In this case, we decided to create a factor with this name, reflecting its definition. Table 9 shows the Learning and Easy of Use’ dimension items.

#### 3.2.10. Efficiency Dimension

The Efficiency dimension assesses whether the product allows users to achieve their goals quickly [27]. This dimension implies checking if it is possible to do more in less time, simply, and allowing flexible use. Verifying whether the product provides shortcuts or present the most used features is a way of assessing efficiency. Table 10 shows the Efficiency’ dimension items.

#### 3.2.11. Feedback Dimension

The Feedback dimension consists of informing the user about their actions or their consequences [25]. However, how feedback is presented to the user can impact UX by influencing how the user perceives the product and how they feels about it. For instance, an interface component loading without indicating how much is left to finish this process can negatively influence the UX. However, suppose the interface displays the percentage that is left for the processing to be completed. In that case, the user can estimate how long the processing is expected to take and decide whether it is worth waiting for it. This aspect can make the experience of using the product more pleasant for the user. Table 11 shows the Feedback’ dimension items.

#### 3.2.12. Value-Added Dimension

The Value-Added dimension refers to the value that the product represents—how important the product is to the user [32]. Evaluating this aspect may indicate that the products are chosen because they reflect or represent important values to the user, i.e., values that the product can satisfy, being perceived as valuable. This perception can contribute to the users’ product choice. Table 12 shows the Value Added’ dimension items.

#### 3.2.13. Satisfaction Dimension

When a user creates expectations about a product, the product must meet and satisfy those needs [33]. The Satisfaction dimension is related to evaluating these characteristics. In this dimension, the focus is two-fold: (i) to verify if the product delivers what it promises, and (ii) if the product meets the user expectation when the interaction occurs. Table 13 shows the Satisfaction’ dimension items.

### 3.3. How to Use UX-Tips

The primary purpose of our technique is to enable a holistic evaluation of UX, i.e., to capture both the application problems and the emotions that these problems evoked in the user. To do so, we developed the technique based on different methods. First, UX-Tips has the characteristics of checklist-based methods, since it presents evaluative items. Evaluative items represent aspects that can be used to verify the adequacy of the application to the items of the technique. Second, our technique is related to form-based methods, which provide a way for the evaluator (the inspector or the user) to describe the problems encountered in order to be able to identify the UX problems.

In this sense, along with the UX-Tips technique, we also created an artifact that contains two fields where the evaluator input the description of their experience: (a) to indicate the technique item’s code to which the problem is associated; (b) to describe the problem. We proposed UX-Tips to be used for both inspection (expert evaluation) and user testing. At inspections, a set of heuristics assists the inspector during the evaluation process to enable the identification of UX problems [34]. The inspector should explore the user interface, considering the evaluative items proposed by our technique.

If the inspector realizes that one of the items is not met, they should fill a form, reporting the item and the problem description from their point of view. On the other hand, during the user testing, users should use the application under assessment and report their difficulties to the moderator, who should conduct the test and is responsible for taking notes and relating the problems with an item of the technique. In order to collect more detailed data from the user, the moderator can ask questions based on the technique items when the user is having difficulty performing a task.

**Figure 2:**
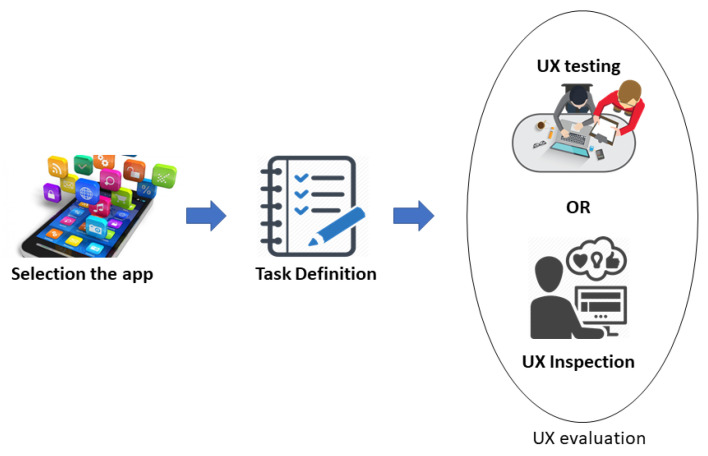
The UX-Tips evaluation process.

Figure 2 presents the steps that should be followed to evaluate the software UX using UX-Tips. First, the evaluator defines the app to perform the evaluation. After defining the app, it is necessary to define the tasks to be performed during the UX evaluation. This step is important to standardize the use of the application by the participants, thus, making them evaluate the same interaction.

**Figure 3:**
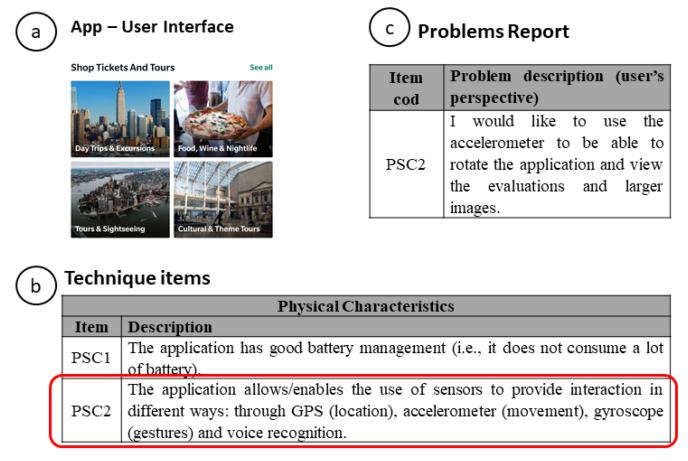
Process to report a problem with UX-Tips. (**a**) Using the app and identification problem step. (**b**) Using the technique to relate the problem and the evaluative item. (**c**) Describing the problem step.

In the evaluation steps, the evaluator will be using an application that will be under evaluation. When interacting with the application, the evaluator will find aspects of the application that they considered problematic. In the case of Figure 3, the problem is related to a need that the user felt to use different resources (e.g., an accelerometer) to improve their user experience. In this sense, the evaluator (in inspection) used a dimension of the UX-Tips technique that assesses the use of hardware to improve the user experience (Physical Characteristics—see Table 6), or the user report the problem to the moderator (in user test) indicating the UX-Tips item related to the problem.

We can see that the diversity of items of the technique allowed the participant to report a specific problem that affected his experience when interacting with the application. The flexibility of UX-Tips does not just refer to the use in the inspection or user testing. We can use it both to evaluate an application already developed, as an application in the development phase. To demonstrate that flexibility, we used UX-Tips in two scenarios. In the first study, we used UX-Tips to evaluate a ready-made application available on the market. In the industry study, we used UX-Tips to evaluate an application in the development phase, helping to identify improvement opportunities before the implementation phase.

**Figure 4:**
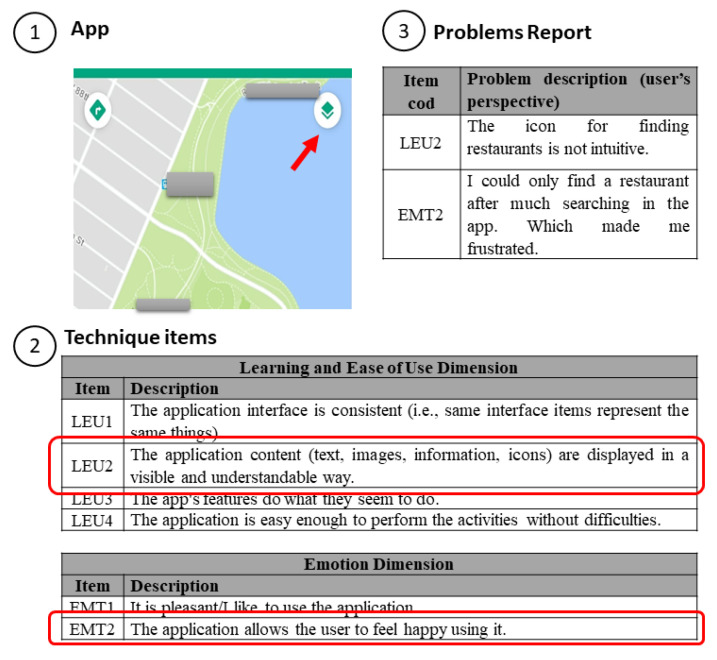
A practical example of using UX-Tips.

We selected another example that shows how the technique allows the collection of both users’ problems and emotions to emphasize identifying problems further and how they affected the user’s experience of use. We present a practical and real example from our results and illustrate the use of UX-Tips by presenting the steps required to perform a UX evaluation. In Figure 4, the problem is related to an icon that does not represent its functionality (finding a restaurant) (see Figure 4—part 1).

Then, the moderator (in user testing) or the evaluator themselves (in inspection) checks in the technique whether there is an item on which they can relate the problem that they encountered (LEU2) (see Figure 4—part 2). Then, they also report how the problem affected their experience (EMT2) through a problem reporting form (see Figure 4—part 3). This data triangulation provides rich data about the UX and shows that UX-Tips allows identifying the problem and how the user felt about it. Furthermore, this example also shows a problem with an icon in the interface, a specific software application characteristic that a general UX evaluation technique may not identify.

## 4. Comparative Study

We carried out the first empirical study to investigate the performance of UX-Tips and to mitigate the lack of comparisons between UX methods [6]. To do so, we compared UX-Tips with the IHI technique. Before selecting IHI, we aimed to compare UX-Tips with other standard measures in UX evaluation (e.g., AttrakDiff and UEQ techniques). However, since AttrakDiff and UEQ are scale-type techniques, and their purpose is to provide a quick evaluation instead of an evaluation with more complete results, we decided not to use them in the comparison to avoid biased results.

Therefore, we defined some criteria to avoid comparing UX-Tips with a very different technique to yield unfair results. The criteria were: (i) to be an inspection technique, with items in a checklist format; (ii) to consider that UX is not only the evaluation of emotional aspects, but the evaluation of all aspects related to the experience (e.g., utility, usability, and emotion), as suggested by Hassenzahl [19]. These criteria resulted in two techniques. In order to obtain more details and the necessary materials to apply them, we attempted to contact the authors of both techniques.

Only the IHI authors sent us the materials. However, IHI does not provide an artifact or a standardized way to report problems. In this regard, we adapted the problem reporting form from UX-Tips so that the participants using IHI can report the problems in the same way participants using UX-Tips do (by providing the ID of the item and the description of the problem).

### 4.1. Participants

We carried out the study with 68 participants. Of those, 54 were from the Federal University of Technology—Paraná (UTFPR) and attended the Human–Computer Interaction (HCI) course. The remaining 14 were part of a Junior Company hosted in UTFPR, which provides services related to consulting and development of Web/mobile systems. They had already taken the HCI course. We selected students from the academy and the Junior Enterprise to form a population with different backgrounds for the study.

### 4.2. Planning

All participants answered a profile questionnaire, which we used to balance the groups and avoid selection bias. The questionnaire had two questions intended to classify the participants regarding two different levels of experience. One of these levels was software development experience in the industry. This was classified as low (L) if the participant had never worked in the industry; medium (M) if the participant had an experience of one year or less; and high (H) if the participant had worked in the industry for over a year.

The other classification was the experience with Usability or UX evaluation. This was classified as high (H) if the participant had already participated in one or more Usability or UX evaluations; or medium (M), if the participant only knew the concept of Usability or UX but had not yet participated in practical evaluations. In this study, no participants were classified with low experience concerning their level of experience with usability or UX evaluations because all the study participants were already introduced to the concepts of usability and UX.

The materials used in the study were the following: (i) a Consent Form; (ii) a profile questionnaire; (iii) a script of activities to be carried out with the application; (iv) one UX evaluation technique (UX-Tips or IHI technique); (v) a form to report the problems encountered, and (vi) a feedback questionnaire applied after the study. The profile and feedback questionnaires were available online to simplify answering and data collection. The script, the two techniques, and the form for reporting problems were available to participants digitally. The Consent Form was printed and delivered to each participant. All the artifacts used in the study are available in the supplementary materials.

In order to compare the two techniques, we chose the TripAdvisor™ (https://www.tripadvisor.com.br/, accessed in 2019) mobile application. TripAdvisor™ is one of the most popular sources of Internet hotel ratings, with more than five million registered users visiting the platform 30 million times a month on average [35]. In addition, the app has versions for both Android and iOS devices. Participants used their own mobile devices in the study.

### 4.3. Execution

We conducted the study in three sessions on three different days due to the number of participants and their availability. At each session, we divided the participants into two groups, where each group used one of the techniques to perform the UX evaluation. All study participants signed the Consent Form and answered the profile questionnaire. Thirty-four participants were allocated for each group, which corresponded to each technique used in the evaluation.

In all sessions, we performed two procedures. The first one was training the participants to know how to use each technique when performing the UX evaluation. This step presented the concepts of usability and UX and the difference between these two approaches. The training included two practical examples of how to proceed to evaluate UX using the techniques. However, to avoid bias in the study, no reference to either technique was made during the training.

The training lasted approximately 15 min. In addition to training, all participants took Usability and UX classes and other UX evaluation methods before the study. The second one was the UX evaluation of the software application by the participants with one of the techniques, according to their assigned group. After completing the UX evaluation procedures, we sent the feedback questionnaire to collect participants’ opinions regarding the UX evaluation technique they used.

To perform the quantitative analysis, we organized all data obtained through the study in a table. An important activity when analyzing an inspection-based evaluation involving multiple evaluators is to bring together all the discrepancies pointed out by the participants in a single list [36]. In this way, we can classify the discrepancies (any report made by a participant in their evaluation) in order to identify those that are duplicated (different discrepancies for the same problem), false positive (discrepancies that do not represent real problems), or real problems in the application.

During the consolidation phase, we grouped discrepancies with similar descriptions (e.g., *“I didn’t find a share button”* and *“there is no share button”*) and account for only one of them, with the other similar ones were counted as repeated (Rep). Similar discrepancies from other participants were considered duplicates and were accounted once per participant in order to obtain their respective effectiveness (e.g., *“I can’t see all the information about the restaurant”* and *“There is a lack of information, when I went to look for information about a certain restaurant, I had problems, such as not finding the average price of some restaurants and meals”*). If the discrepancy did not represent a real UX or application problem, we classified it false-positive (FP).

Two researchers analyzed the discrepancies at the discrimination meeting, where the researchers analyzed the classifications until they reach a consensus of all discrepancies. After the analysis, a total of 120 unique problems were obtained, where 69 problems were identified only by UX-Tips, 34 were identified only by IHI, and 17 were captured by both techniques.

### 4.4. Analysis

We conducted different forms of data analysis. First, we performed a quantitative analysis of efficiency and efficacy. These metrics are important because they provide an overview of how many problems an evaluator can find in a specific unit of time, such as an hour (efficiency) and the percentage of problems encountered by an evaluator (efficacy) using a specific technique. Therefore, we aimed to test the following hypotheses:

- H01: There is no difference in terms of efficiency between UX-Tips and IHI techniques.
- HA1: There is a difference in terms of efficiency between UX-Tips and IHI techniques.
- H02: There is no difference in terms of efficacy between UX-Tips and IHI techniques.
- HA2: There is a difference in terms of efficacy between UX-Tips and IHI techniques.

We defined Efficiency as the ratio of the total amount of defects found by all participants to the total amount of time required in the inspection process. To obtain Efficacy, we first calculated the Efficacy obtained by all participants individually, which is the ratio of the number of problems found individually by the total amount of problems found in the application. Then, we calculated the mean of the individual Efficacy to obtain the Efficacy of the techniques. These definitions are following previous studies conducted in usability or UX evaluations [37].

To test the hypotheses, we performed statistical analysis by using IBM SPSS v24 tool (https://www.ibm.com/br-pt/products/spss-statistics, accessed in 2019) to verify whether there was a significant difference between the results from each method per indicator. Before running a statistical test, we needed to know how the data distribution, given that different experiment designs and data distribution require different statistical tests [38]. To do so, we performed a Kolmogorov–Smirnov (sample size > 50) normality test to select the adequate statistical test. If *p*-value > 0.05 (i.e., the distribution is normal) in both groups for a given indicator, we applied Student’s *t*-test. On the other hand, if the *p*-value < 0.05 (i.e., the data does not follow a normal distribution) in at least one group for that indicator, we applied the Mann–Whitney non-parametric statistical test. We also calculated the Effect sizes at a 95% confidence interval using Cohen’s d [39].

After the quantitative analysis, we conducted a Summative Content Analysis (SCA) [40], which involves counting and comparisons, usually of keywords or content, followed by interpretation of the underlying context. In this analysis, the quantification is an attempt not to infer meaning but, instead, to explore usage [40]. We did the SCA with two goals. First, the descriptions of the problems provided by the participants were used to obtain additional information about the experience, complementing the UX evaluation. Second, the responses to the feedback questionnaire were observed to evaluate their perception of the techniques used.

We categorized the participants’ responses into three different categories: (i) positive, if the participant answered “yes” or indicated positive aspects of the techniques, (ii) negative, if the participants answered “no” or indicated problematic aspects of the techniques, and (iii) “neutral” if the participants indicated that the techniques partially assisted.

### 4.5. Results

#### 4.5.1. Quantitative Data Analysis

Table 14 presents an overview of the total number of discrepancies reported (Discr.), the number of false positives (FPs), duplicate (Dupl.), i.e., different discrepancies that represent the same problem, repeated (Rep.), i.e., same discrepancies reported in different script activities, and the number of unique problems (UP). We see that the number of discrepancies reported by UX-Tips was higher than IHI. Therefore, UX-Tips enabled participants to find more problems (UP). This may be explained by the fact that, although the techniques have a similar number of items, the dimensions proposed in UX-Tips may lead to the search for problems outside the scope of IHI’s dimensions. Notice that this also happens in the opposite direction. IHI has some dimensions out of the UX-Tips scope, but to a smaller extent.

We analyzed the discrepancies classified as FPs to better understand what the number of FPs meant in each technique, given that this amount was considerably different in each one. Regarding the six FPs occurrences in the UX-Tips, we identified that participant P42 misunderstood the evaluative item VLE2 (The application has/represents values that are important to the user), answering it ambiguously. Participant P42 responded to this item: *“Yes, several unnecessary, and what I am looking for, it does not show”*.

Considering this report, it is not clear exactly what the participant refers to as “unnecessary” and “does not show”. Thus, we consider this report as FP. Two discrepancies were classified as not real problems in the app (e.g., *“During the execution of this task, I noticed two options leading to the same destination”*). Three other discrepancies were classified as issues external to the reviewed app (e.g., *“The app makes me feel a little uncomfortable due to high prices”*). In the case of TripAdvisor, the purpose of the app is to inform and not establish prices charged by establishments.

Regarding the IHI technique, three FPs were misinterpreted items of the technique, twenty-two were considered non-problems in the application, and the other 16 FPs were considered positive aspects reported during the evaluation *(e.g., “I found no challenge. It was easy to use all the options”*). We counted positive aspects of the application as FPs since the purpose of the evaluation was to identify problems. The positive reports indicate which aspects of the application do not have UX issues, and thus they do not necessarily point to improvement aspects.

As for repeated discrepancies (see Table 14—Rep.), 20 occurrences were registered in UX-Tips and 33 in IHI. We observed that the issues considered as repeated recur at various points in the user’s interaction with the app during the analysis, causing the user to report the issue multiple times. In this sense, we consider it as an opportunity to improve the technique. In the Problem’s Report artifact, we can insert a field for the evaluator to report that the problem is persistent throughout the app or in part, reducing inspectors’ time in the problem consolidation stage. Regarding duplicates, UX-Tips had more occurrences than IHI.

This may indicate that the UX-Tips technique led participants to find out the same problems. These results show that, besides finding more problems, participants using the UX-Tips technique made fewer errors (by finding the same problems) than those who used the IHI technique. For instance, we took the most reported problem in each technique to analyze how many participants found the same problem.

At UX-Tips, the problem related to finding a restaurant in the app was the most recurrent, with 35 occurrences among 25 different participants. At IHI, the most recurrent problem was that the application did not have an initial guide for use. This issue was reported 14 times by 11 different participants. With these two examples, we can see that the problem most reported through UX-Tips is more practical, focused, and related to the proposal of the evaluated application. At IHI, the most reported problem indicates a more technical issue, a general problem in any application. This could indicate that the UX-Tips allows a more focused assessment in context. However, a more in-depth analysis is needed in this regard.

**Figure 5:**
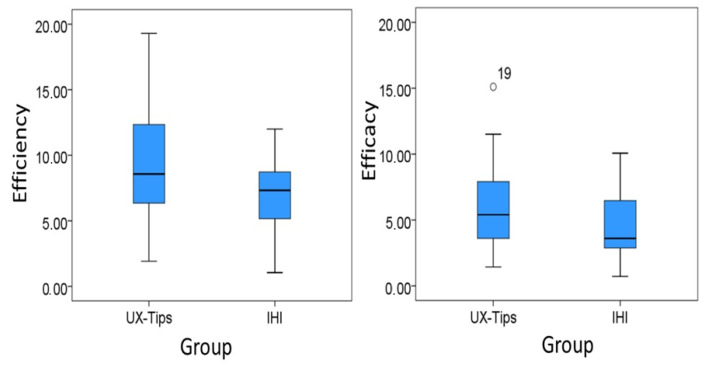
Boxplots for efficiency and efficacy.

As Figure 5 shows, UX-Tips’ group median is almost at the same level as IHI. We used the Kolmogorov–Smirnov test to test the normality since the sample was larger than 50. We verified that efficiency was normally distributed in both groups (*p*-value = 0.162 for UX-Tips and *p*-value = 0.200 for IHI). In order to determine whether the difference between the samples is significant, we applied the parametric *t*-test for independent samples [41]. When we compared the two samples using the *t*-test, we found significant differences between the two groups (*p*-value = 0.003). These results support the rejection of the null hypothesis H01 (*p*-value < 0.05), and the acceptance of its alternative hypothesis HA1, suggesting that UX-Tips was more efficient than IHI when used to find UX problems in this experiment.

In order to evaluate the effect size, we computed Cohen’s d. Considering Cohen’s convention, the effect size for efficiency was medium (d = 0.76) [39]. The mean difference was 2.74 defects/hour, i.e., UX-Tips participants found from two to three more defects than IHI participants for each hour dedicated to the UX evaluation task.

The boxplot graph with the efficacy distribution per technique suggests that UX-Tips’ group was more effective than IHI’s group. The UX-Tips’ group median was higher than the IHI’s group median. The number 19 in Figure 5 represents the participant who had the best performance in this indicator related to UX-Tips. Despite this participant’s low experience in the industry and medium experience with UX and usability, all discrepancies he reported were real problems. We did not find any particular characteristic that could explain this superior result, comparing to other participants. According to the course instructor, he was a highly engaged student.

We also used Kolmogorov–Smirnov to test the normality. We verified that efficacy was not normally distributed in either group (*p*-value = 0.014 for UX-Tips and *p*-value = 0.000 for IHI). In order to determine whether the difference between the samples is significant, we applied the Mann–Whitney test [42]. The *p*-value obtained in the Mann–Whitney test was 0.044. This result, therefore, supports the rejection of the null hypothesis H02 (*p*-value < 0.05) and the acceptance of its alternative hypothesis HA2, suggesting that the UX-Tips technique was more effective than IHI when used to find UX problems in this experiment.

The effect size for efficacy was also medium (d = 0.52) [39]. The mean difference was 1.48%, i.e., the percentage of defects UX-Tips participants found from the total existing ones was 1.48% higher than the percentage of IHI participants.

Summary of quantitative analysis: Participants using UX-Tips reported more problems than participants using IHI and, therefore, were more effective. This may be explained by the more significant number of unique problems they found. They were also more efficient than participants using IHI, which may be related to their higher precision during the evaluation. Given that they had a smaller number of false positives, they may have spent less time finding real problems.

#### 4.5.2. Summative Content Analysis

We divided the Summative Content Analysis (SCA) into two parts. The first part is related to the results obtained by technique, which allow the participants to describe the problems they encountered. The second part is related to the analysis of the answers gathered from the feedback questionnaire, which evaluated the perception of the use of the techniques.

In the first part, we looked at how the problems encountered by the participants affected their UX. Table 15 presents reports of statements about the main problems they identified, associating them to the technique used by the participant.

As Table 15 shows, almost all participants that described the consequences of the problems to their experience with the app were from the UX-Tips group. This may indicate that the description of IHI items may not evaluate hedonic attributes explicit to the participants. Thus, by allowing participants to describe their experiences, UX-Tips can aid them in expressing how a problem can affect UX. This characteristic may be necessary for the design team to verify the causes for a negative UX.

The second part of the SCA is related to the user perception of the techniques through the answers gathered from the feedback questionnaire. Since this questionnaire was available online, some participants did not respond to it (33 participants used UX-Tips, and 29 used IHI). Regarding the feedback questionnaire, Table 16 presents the distribution of positive (Pos.), negative (Neg.), and neutral (Neu.) answers, i.e., the technique (Tech.) partially helped. The participants answered balanced regarding the appropriateness of the techniques and their ability to describe the problems that they found.

Considering that one of the objectives of UX-Tips is to allow the identification of UX problems, it was noteworthy that most participants answered that UX-Tips enabled indicating UX problems, confirming the results in Table 15. The same may not be true for IHI. Although the number of participants using IHI also indicates that it enabled the description of problems (considering we use the UX-Tips problems form to IHI), Table 15 shows an example that few participants reported the details about the problems they found.

Regarding ease of use, Table 16 shows that most of the participants using the UX-Tips technique affirmed that it was easy to use. On the other hand, the number of participants that indicated that using IHI was easy to use was almost the same as the number that indicated that it was not easy to use. The difficulty in using might explain the high number of FPs in IHI (see Table 14). Below, we highlight statements of ease of use of both techniques to present more details on their perceptions about this topic.

“It was easy. The descriptions were well didactic as to what to do.”

—(UX-Tips)

“Yes, the items were very self-explanatory, more direct on the tasks to be performed and questions to be found in the application.”

—(UX-Tips)

“It was easy to use the technique and understand it.”

—(IHI)

“It was not easy, some items were unclear and could be interpreted differently.”

—(IHI)

Summary of Summative Content Analysis: Participants who used IHI did not describe how problems affected their experience, limiting themselves to point out the problem without detail. Furthermore, UX-Tips encouraged participants to report their feelings regarding the problems, showing that the technique aids in assessing the hedonic attributes. Lastly, UX-Tips had a higher proportion of participants regarding the ease of use when compared to IHI.

## 5. Industry Study

With the positive results of the comparative study, we decided to evaluate the suitability of the UX-Tips technique to the industrial setting. The participants used UX-Tips to evaluate a high-fidelity prototype of a mobile application under development.

### 5.1. Company and Participants

The company where we carried out this study was a mixed economy one that started to operate in 1972 located at Manaus city. The company provides Information Technology (IT) services, including the development of information systems and websites. Their primary clients are the units of the State Public Administration, although they may also provide services for public units at other levels, such as federally and to private organizations.

In this study, the participants evaluated the prototype of a mobile application referred to as Application X due to confidentiality constraints. The prototype was composed of screens and their interaction designed to be part of the final product. The application aims at providing citizens easy access to varied information about public units and services. For instance, if a citizen wishes to get a driver’s license, they can use Application X to consult what public unit to contact and what documents are needed as well as to schedule the service to obtain it.

The evaluation involved five inspectors and ten end-users. Two of the inspectors were employees from the company, and we checked their expertise in usability and UX evaluations before the evaluation session. The remaining three inspectors were researchers either of HCI or Software Engineering and were highly experienced in UX and usability. The participating end-users were potential users of the application, given that they were common citizens. They had varied work backgrounds, but only one of them worked in the IT field. We verified their level of experience regarding the use of technology in general, including their ability to use computers and mobile smartphones.

### 5.2. Planning

In this study, we aimed at analyzing the use of UX-Tips in an industrial context through the evaluation of Application X. We considered two measures—efficacy and efficiency—and two different evaluation approaches—inspection and user testing. We collected feedback from participants about their perceptions of the technique. In user testing, we selected participants from the set of users of the company. To guide the evaluation, we delivered them an activities script. We used the concept of personas to create an activity script. We identified two personas for Application X during this task, and four activities were devised to compose the activities script. Details about the personas and the activities script are given in the supplementary material.

### 5.3. Execution

The industry study had two rounds. The first involved inspectors only (P01—P05 in Table 17), who were trained to use UX-Tips before the inspection. The training lasted approximately 20 min and was held in a single session with all inspectors. Next, inspectors were introduced to the Application X prototype. Then, they executed the inspection process, taking notes about their starting and finishing times. This data was later used to calculate inspectors’ efficiency. In the end, inspectors had to answer a feedback questionnaire.

The second round of the study involved ten end-users (P06—P15 in Table 18) from the participating company. Each end-user evaluated Application X individually with the support of one moderator. Moderators had the responsibility to present Application X to the participant and information about how to evaluate UX using UX-Tips. Participants also took notes about their starting and finishing times to allow the calculation of their efficiency.

### 5.4. Results

As we proceeded in the comparative study, we organized all discrepancies into one single list. The classification of discrepancies followed the same rules that we established in the comparative study presented in Section 4.3. In addition, the rating validation was also performed with the review of a UX expert.

This section presents the study results in terms of efficiency and efficacy considering both the inspection and user testing. In this study, we use the same definitions of efficiency and efficacy as the first study. Table 17 presents the efficiency and efficacy, as well as the experience level of each inspector, and Table 18 presents the exact data for users.

Another essential aspect for enabling the correct interpretation of efficiency and efficacy results in this study was the definition of an oracle. We analyzed all discrepancies reported, selecting all the unique problems (UP) found both in inspection and user testing to compose the oracle. There were 64 unique problems in total: 26 were found during inspection, 24 were found during user testing, and 14 were found in both evaluation activities.

Table 17 shows that only one inspector, P03, reported discrepancies later classified as false positives since they were not real problems of Application X. Instead, they were positive aspects the inspector considered important to highlight. Although the objective of UX-Tips is to allow the identification of UX problems, i.e., negative aspects, this occurrence may point to another use for UX-Tips. Table 18 also shows that four different users found false positives—i.e., less than half of participants reported discrepancies that were not real problems. As with the inspection, false positives here are related to the positive aspects of Application X. We consider this a positive result regarding the use of the technique, indicating it is helpful in guiding inspectors and users in identifying real problems.

Another piece of data that stands out is the number of problems found by inspector P01. By analyzing the discrepancies reported by this inspector, we found that they presented a highly detailed evaluation. The P01 inspector used both pragmatic and hedonic items from the proposed technique to report different aspects of the same problem. For instance, in one of the activities in the script, the inspector had to choose one city. In the application, the inspector had to select the chosen city and then click on a confirmation button. The inspector considered the confirmation step unnecessary and reported this as a problem using efficiency dimension, item EFF2 (the application allows using shortcuts to perform some activities), related to a pragmatic item.

However, the inspector also reported that selecting something and then confirming it was stressing, using the emotion dimension, item EMT2 (the application allows the user to feel happy using it), which is a hedonic one. This occurrence shows that UX-Tips allows the user to indicate the problem and express how it affects him emotionally.

Table 19 presents the results for UX-Tips regarding efficiency and efficacy during the inspection and user testing rounds. One can notice that inspectors were more effective than users when using UX-Tips. However, there were two-times more users than inspectors, and P06 and P13 identified only three problems. Thus, their efficacy was equal to 4.69%—much less than the mean for the total number of users, which was 11.56% as Table 19 shows. The means for efficiency were more balanced between the two groups. Inspectors were slightly more efficient than users. However, user testing sessions were moderated, and the time counting also included the interaction between moderators and users.

Another result from the industry study is related to the answers given by inspectors to the feedback questionnaire. All inspectors affirmed that UX-Tips enabled them to evaluate the user experience, positively answering the first question. Four of them answered positively about whether UX-Tips enabled them to indicate the UX problems they found. One of them was not sure if that was the case. He had doubts about whether he should report any variation in his satisfaction during the evaluation session. Regarding easiness of use, three inspectors considered the technique easy to use, one of them considered it partially easy, and one of them regarded it as hard to use.

Through the feedback questionnaire, inspectors also gave suggestions about how to improve the technique. They highlighted the need to include the report of positive aspects about the products with the UX-Tips (P04—“one suggestion would be a version to indicate the positive UX factors perceived in the application.”) as well as the need to customize items since some of them may not be relevant to some applications. Another suggestion was the creation of two sections in the technique description, separating the items that focus on the evaluation of the general perception (P05—“One suggestion would be to place the items that evaluate the overall perception of the application in a separate section where the inspector will evaluate these items after completing the inspection.”), so that they may be used at the end of the interaction with the application (i.e., satisfaction).

## 6. Discussion

This section presents the main findings that we identified during the studies carried out to test the UX-Tips’ efficiency and efficacy. The results revealed that UX-Tips obtained better indicators in the UX evaluation performed in the comparative study. UX-Tips allowed participants to be more effective in identifying UX issues. By allowing users to find more unique problems, UX-Tips contributes to a holistic improvement of the evaluated application. In addition to finding more problems, a technique must have a good cost–benefit relationship—that is, finding more problems in the shortest time possible. UX-Tips also had better efficiency indicators compared with IHI, meaning that participants who used UX-Tips in the assessment took less time to find more problems.

The FPs analysis of the two techniques allowed us to identify an interesting finding for research in UX, specifically in the development of new methods. In the comparative study, IHI led participants to report some positive aspects when using the app evaluated. In the results obtained from the industry study, we also verified in inspection that UX-Tips was used to report positive aspects of the evaluated application (inspector P03). Considering the holistic characteristic of UX, it might be interesting to collect positive feedback from experience and report them in order to help in different research perspectives.

For example, it is possible to build development patterns from what users find positive about software applications to help developers and industry professionals design applications with a good UX. Thus, we can add more value to the industry and add contributions that complement RQ2’s response on meeting industry goals when considering UX.

Despite the positive aspects arising from the FP analysis findings, many FPs are not interesting in the industry context. To point out a FP, one spends analysis time that does not indicate a real problem. In this sense, the UX-Tips allowed participants to report more real problems in the comparative study, implying a lower incidence of FPs than IHI. The industry study supports this result, where less than half of users reported FPs during user testing, meaning most discrepancies were real issues. This result is significant for the industry, as it allows finding real problems during a UX assessment, making the assessment cost-effective and even contributing to the UX assessment being more adopted in the industrial context.

Another finding supported by the comparative and industry study is that participants reported that UX-Tips allows them to express the feeling evoked when faced with an application problem. That means they report both the problem and the feeling that the problem caused in the experience. This fact shows that the results not only indicate how the UX was from the hedonic perspective (that is, how the user felt when using the application) but also allow identification of the possible problems that may have led to a bad feeling, causing a negative UX. In addition to allowing a holistic understanding of the experience, this aspect of UX-Tips can facilitate application improvement by the development team.

Regarding the study in the industry, it is possible to verify that the number of problems identified through inspection (26) and test (24) are practically the same and refer to different identified problems since they were considered unique problems. This balance shows the importance of considering both approaches to obtain an assessment with good coverage of issues. Thus, it is essential that a technique allows its use from both approaches, as the UX-Tips does. This result also allows us to infer the ease of use by common users since the technique allowed them to find almost the same number of problems that the inspectors found.

Bellow, we present the contributions of our technique, relating its benefits to the limitations found in the literature. To discuss our results, we will return to the research questions defined in Section 1 to show how the results obtained in this investigation help to answer them.

RQ1. Does UX-Tips allow users to report issues that caused a negative UX?

UX-Tips allows users to report their experiences exactly how they want to express themselves through a form to report the problems. The description of the problems in this form helps understand how users described a problem and how they felt about it. Results from different studies have identified that scales are the most commonly used methods to evaluate UX [1,6,8]. However, as shown in Marques et al. [7], this method does not produce detailed results to help to understand what caused a negative UX holistically.

Based on the results of the studies, we can conclude that UX-Tips allows identifying the problems that caused a negative UX. For instance, participant P37 used a pragmatic and a hedonic item to indicate the difficulty of finding information on TripAdvisor. They used the LEU4 item (ease of use) to indicate that the information was not easy to find, and used the EMT2 item (checks the user’s happiness when using the app) to indicate that they were not happy about it. Thus, providing a means to guide the assessment and a way for users to report how they felt allows collecting rich data about UX. This helps to provide more valuable results than simply indicating whether UX was negative or positive, without identifying what can be improved. This feature makes our technique stand out from existing ones. It is fundamental to understand what to improve in order to provide a good UX.

Furthermore, Bargas-Avila and Hornbæk [6] stated that the proposals of new UX techniques are rarely validated and compared. This paper showed a comparison between UX-Tips and others considering several measures, such as efficiency, efficacy, identified problems, false positives, and the participants’ opinion. These measures strengthen the comparison between the two techniques and allow a more in-depth analysis of each one, in addition to serving as guidelines for new comparisons within UX research.

RQ2. How does UX-Tips meet industry interests when evaluating UX?

In Section 2, we presented some characteristics that make a UX technique attractive to practitioners, i.e., for application in industry settings. We discuss these characteristics by presenting how UX-Tips can address them.

Roto et al. [24] identified that, in industry, the main requirement for UX assessment methods is that they are inexpensive (i.e., do not require many resources), are quick, and are simple to use. In this sense, UX-Tips does not require many financial resources: it can be printed on paper, reducing the evaluation cost. In addition, our results showed that UX-Tips could be used in an industry setting by both inspectors and end-users (with the help of a moderator). In both cases, problems could be found not investing more than one hour by inspection and twenty minutes through user testing. These findings show the UX-Tips’ ease of use and feasibility.

Another critical feature for UX assessment techniques to be of higher utility to the industry is that they enable a qualitative assessment [10,11] as this approach instigates identifying information relevant to the application design. UX-Tips was designed to identify UX issues and gain user insight into how UX issues might have affected UX. Thus, in addition to allowing a quantitative analysis of the UX evaluation, UX-Tips enables a qualitative analysis through the descriptions provided by the users.

Lallemand et al. [10] identified that the industry’s primary interest in UX is the possibility to design better products, and practitioners are looking for a definition that considers various aspects of interaction. By supporting the identification of UX problems, UX-Tips allows improving the app’s quality by addressing these problems. UX-Tips has a deep theoretical foundation and considers various dimensions of UX collected in the literature, showing the breadth of evaluation through UX-Tips.

## 7. Limitations

Regarding the comparative study, one limitation is using a single technique as the basis for the comparison. However, instead of choosing many different techniques, we chose one covering a wide range of UX dimensions, allowing us to keep the study cost-effective. Furthermore, we established criteria to choose the technique as presented in Section 4.

Another limitation in this study is confounding factors, such as the participant differences in performing tasks, such as the ones proposed in the experiment. To address this issue, we balanced the groups, given the participants’ experience with usability or UX evaluations and in software development in the industry. Both groups were trained in the UX evaluation techniques before the experiment, which may lead to training effects. To address this, we provided both groups with the same training. The training material included examples from both techniques without naming any of them to avoid bias.

Finally, another limitation is related to the way problems are reported using the IHI technique. There is no standard artifact for reporting problems using the technique. In order to avoid the results of the IHI technique being harmed by the lack of artifact, we decided to use the same problem reporting artifact from the UX-Tips technique, making it clear during training how to use it to report problems in both techniques.

## 8. Conclusion and Future Work

In this article, we presented UX-Tips, which aims to provide valuable results in a UX evaluation by indicating the issues that may be considered for improving an application. We performed two studies in two different settings (academic and industrial) to analyze our technique. Our results revealed that UX-Tips allowed identifying UX problems and met industry and professional interests when evaluating the UX of products. Additionally, UX-Tips was considered easy to use, did not require many resources, and contributed to understanding what may have caused a negative UX. These aspects meet the needs raised in the literature for the development of UX techniques.

As future work, due to the substantial amount of research in the UX field, we intend to investigate recent work, such as Petterson et al. [1] in order to include new UX dimensions. For instance, we discussed that UX is subjective and contextual; therefore, it is necessary to consider the specifics of each context when evaluating UX. Technology advancements have allowed the development of different software applications that have changed the way users interact and consume content through software systems. Increasingly, there is the emergence of applications aimed at entertainment and interaction in immersive contexts [43], thus, introducing immersion with a new form of user interaction and bringing new dimensions to assess the UX, such as presence, flow, and immersion.

Pettersson et al. [1] identified a growing interest in evaluating the UX of immersive experiences, such as the experiences provided by Virtual Reality, and it is necessary to develop UX evaluation methods for this context. We intend to introduce the UX dimensions aimed at immersive experiences in UX-Tips to address this category of software application. We intend to evaluate the use of UX-Tips in other types of applications, such as websites, to verify the suitability to evaluate the UX of different software applications.

## Figures and Tables

**Table 1:** The Aesthetics dimension and its items.

Dimension	Code	Item Description
Aesthetics	AST1	The application features a nice and beautiful interface design.
	AST2	The color and contrast scheme shown is appropriate.

**Table 2:** The Emotion dimension and its items.

Dimension	Code	Item Description
Emotion	EMT1	It is pleasant/I like to use the application.
	EMT2	The application allows the user to feel happy using it.

**Table 3:** The Engagement dimension and its items.

Dimension	Code	Item Description
Engagement	EGT1	The application arouses the interest in obtaining it.
	EGT2	The application stimulates the desire to recommend it to others.
	EGT3	The application stimulates the curiosity to know it more.

**Table 4:** The Innovative dimension and its items.

Dimension	Code	Item Description
Innovative	INO1	The application has innovative features (different ways to meet the user’s needs).

**Table 5:** The Social dimension and its items.

Dimension	Code	Item Description
Social	SOC1	The application lets you share information with others.
	SOC2	The application allows being always updated (aware) about the content it provides.
	SOC3	The application is known and widely used by other people.

**Table 6:** The Physical Characteristics dimension and its items.

Dimension	Code	Item Description
Physical Characteristics	PSC1	The application has good battery management (i.e., it does not consume a lot of battery).
	PSC2	The application allows/enables the use of sensors to provide interaction in different ways: through GPS (location), accelerometer (movement), gyroscope (gestures) and voice recognition.

**Table 7:** The Utility dimension and its items.

Dimension	Code	Item Description
Utility	UTL1	The application assists in an important activity.

**Table 8:** The Control dimension and its items.

Dimension	Code	Item Description
Control	CTR1	The application allows controlling the interaction the way the user wants.

**Table 9:** The Learning and Easy of Use dimension and its items.

Dimension	Code	Item Description
Learning and Easy of Use	LEU1	The application interface is consistent (i.e., same interface items represent the same concepts).
	LEU2	The application content (text, images, information, and icons) are displayed in a visible and understandable way.
	LEU3	The app’s features do what they seem to do.
	LEU4	The application is easy enough to perform the activities without difficulties.
	LEU5	The application visibly provides tips or guides on how to use it.
	LEU6	The application does not require much mental effort to remember how to use it.

**Table 10:** The Efficiency dimension and its items.

Dimension	Code	Item Description
Efficiency	EFF1	The application processes the information quickly.
	EFF2	The application allows using shortcuts to perform some activities.

**Table 11:** The Feedback dimension and its items.

Dimension	Code	Item Description
Feedback	FDK1	The application provides information about the actions the user performs.
	FDK2	Information about user actions is objective and understandable.

**Table 12:** The Value Added dimension and its items.

Dimension	Code	Item Description
Value-Added	VLE1	The application generates value (has benefits that make the user prefer this application over the competitors).
	VLE2	The application has/represents values that are important to the user.

**Table 13:** The Satisfaction dimension and its items.

Dimension	Code	Item Description
Satisfaction	STF1	The application meets user’s expectations.
	STF2	The application fulfills what it is expected to do.

**Table 14:** Performance by technique.

Technique	Discr.	FPs	Dupl.	Rep.	UP
UX-Tips	320	6	208	20	69
IHI	285	41	158	33	34

**Table 15:** Reports about some of the problems found.

Statement	Technique
**Problem: difficulty to find a restaurant near an attraction.**	
“It caused some irritation in trying to find the area where my attraction was to be able to find a restaurant”	UX-Tips
“I was only able to take action [find a restaurant] after much searching in the app. That frustrated me”	UX-Tips
“The user feels discouraged to continue searching for nearby places”	IHI
**Problem: lack of information about restaurants.**	
“No [he wasn’t happy] because the chosen restaurant did not provide prices for some things in the app.”	UX-Tips
“No [the app does not deliver as expected] because it did not provide all the necessary or desired information.”	UX-Tips
“In this task [the search for restaurants] the application could stimulate the curiosity to know it more because the restaurants could be described in more detail.”	UX-Tips
“Not finding information does not make me happy. I would have no problem browsing in a concurrent application.”	UX-Tips
**Problem: lack of initial guides.**	
“It was frustrating not to be able to do one of the script’s activities.”	UX-Tips

**Table 16:** Distribution of the user perceptions by technique.

Question	Tech.	Pos.	Neg.	Neu.
Q1—Is the technique appropriate to evaluate UX?	UX-Tips	30	1	2
	IHI	22	3	4
Q2—Did the technique enable the description of problems?	UX-Tips	23	4	6
	IHI	21	5	3
Q3—Is the technique easy to use?	UX-Tips	22	7	14
	IHI	14	12	13

**Table 17:** Data about the inspectors’ results.

Inspector ID	P01	P02	P03	P04	P05
Experience	High	High	Medium	High	Low
Total problems	23	12	8	18	7
False-Positives	0	0	7	0	0
Time (min)	67	63	65	44	55
Efficiency (defects/hour)	20.6	11.43	7.38	24.55	7.64
Efficacy (%)	35.94	18.75	12.50	28.13	10.94
Efficiency (x)	14.32				
Efficacy (x)	21.25				

**Table 18:** Data about the users’ results.

**User ID**	**P06**	**P07**	**P08**	**P09**	**P10**
Experience	Medium	Medium	High	Medium	Low
Total problems	3	13	7	8	13
False-Positive	0	0	0	1	0
Time (min)	30	29	30	24	30
Efficiency (defects/hour)	6	26.9	14	20	26
Efficacy (%)	4.69	20.31	10.94	12.5	20.31
**User ID**	**P11**	**P12**	**P13**	**P14**	**P15**
Experience	Low	High	High	Low	Medium
Total problems	8	7	3	6	7
False-Positives	0	2	2	1	0
Time (min)	35	61	37	27	40
Efficiency (defects/hour)	13.71	6.89	4.86	11.11	10.50
Efficacy (%)	12.5	10.94	4.69	7.81	10.94
Efficiency (x)	13.99				
Efficacy (x)	11.56				

**Table 19:** The efficiency and efficacy.

	Inspection	Test
Efficiency (defects/hour)	16.73	13.99
Efficacy (%)	21.25	11.56

## Data Availability

The data presented in this study are available in supplementary material here.

## Supplementary Materials

The following are available online at https://www.mdpi.com/article/10.3390/s21217183/s1.
