# Supplementary material for: Understanding UX Better: A New Technique to Go beyond Emotion Assessment
Source: Sensors (Basel). 2021 Oct 29;21(21):7183. doi: 10.3390/s21217183 (PMC8586993; doi:10.3390/s21217183)
Supplement: Supplementary file 1 [file sensors-21-07183-s001.zip › sensors-1403329-supplementary.pdf]

Support Material for UX-Tips Technique Studies

Leonardo Marques, Patrícia Matsubara, Walter Nakamura, Bruna Ferreira, Igor Wiese, Bruno Gadelha, Luciana Zaina, David Redmiles and Tayana Conte
{lcm, patriciagfm, walter, bruno, tayana}@icomp.ufam.edu.br
bruna.antonelli2@gmail.com
igor.wiese@gmail.com
lzaina@ufscar.br
redmiles@ics.uci.edu

USES – Usability and Software Engineering Research Group
Postgraduate Program in Computing
Federal University of Amazonas
Manaus AM, 69077-000

USES
Usability and Software Engineering Research Group

USES Technical Report

Number TR-USES-2020-0004

May 2020

Postgraduate Program in Computing

Federal University of Amazonas (UFAM)

Manaus, Amazonas 69077-000

URL: http://www.ufam.edu.br

Support Material for UX-Tips Technique Studies

Leonardo Marques, Patrícia Matsubara, Walter Nakamura, Bruna Ferreira, Igor Wiese, Bruno Gadelha, Luciana Zaina, David Redmiles and Tayana Conte
{lcm, patriciagfm, walter, bruno, tayana}@icomp.ufam.edu.br
bruna.antonelli2@gmail.com
igor.wiese@gmail.com
lzaina@ufscar.br
redmiles@ics.uci.edu

USES – Usability and Software Engineering Research Group
Postgraduate Program in Computing
Federal University of Amazonas
Manaus AM, 69077-000

Federal University of Amazonas, Postgraduate Program in Computing, TR-USES-2020-0004
May 2020

Abstract: This technical report presents the artifacts we used in two empirical studies. In the first one we compared UX-Tips with IHI technique in order to access which of them is more efficient and effective on identifying UX problems. In the second study, we investigated the use of UX-Tips in an industrial setting by evaluating a real application under development. In order to provide all the details regarding the studies we have performed, we also include a table containing the data of each participant of the comparative study.

Keywords: user experience, ux, ux technique, ux evaluation, industrial study, comparative study.

1 Introduction

In this technical report (TR), we provide the artifacts we have used in some studies to evaluate the UX-Tips technique. In addition to the artifacts, we also included a table containing the raw data of the comparative study. Bellow we describe each section present in this TR.

Section 2 presents the complete UX-Tips technique. The first part contains some instructions on how to perform a UX evaluation using UX-Tips. These instructions are part of the technique and aimed to help users who used UX-Tips.

Section 3 presents the Integrative Heuristic Inspection (IHI) technique, which we used in a comparative study with UX-Tips. We present all the dimensions of IHI and their respective items, as well as their descriptions.

In Section 4, we present the form we developed in order to provide a standardized way for users who used UX-Tips to report the problems they faced and their experiences. Since IHI do not provide a specific way for this, we used the same form from UX-Tips.

Section 5 presents the activity script that we used to guide the inspection of the participants during the comparative study. This script aimed to guide the participants and also to standardize the activities they should follow during the study.

We present, in Section 6, the Feedback Questionnaire we used to get participants' perception regarding both UX-Tips and IHI techniques in the comparative study. We also used this questionnaire to get the perception of the participants of the industry study regarding UX-Tips.

In section 7, we present the details regard the personas we defined using some guidelines of the PATHY technique. We used the personas to develop the activity script of the industry study.

Finally, in Section 8, we provide the raw data of the comparative study in two tables. The first one is related to the participants who used the UX-Tips technique and the second one is related to the participants who used the IHI technique.

2 UX-TIPS Technique

Let's evaluate the **User eXperience** of the ____________ app! To do so, you will use the **UX-Tips technique** that is on the next page.

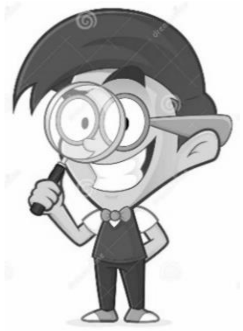

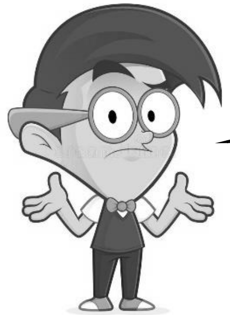

How to **use** the technique's items **to evaluate** the User eXperience?

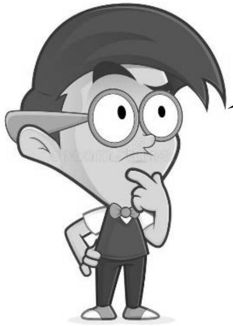

The items represent positive aspects that the evaluated application **should have**. When the application **does not** have a feature related to a given item, this **should be indicated** as a **negative aspect** of the application. See the example below:

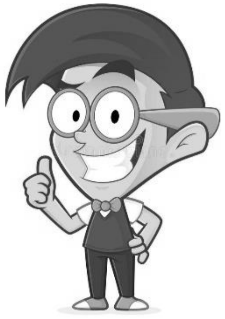

Item **AST1** means: "The application features a nice and beautiful interface design".

But by using it, I am indicating that the application interface is **NOT** nice, as the way the elements are presented is disorganized, **violating** the AST1 item.

Note: If you think a problem may be related to more than one technique item, please indicate the items in the Problem Table.

Aesthetic Dimension	
**Item**	**Description**
AST1	The application features a nice and beautiful interface design.
AST2	The color and contrast scheme shown is appropriate.

Emotion Dimension	
**Item**	**Description**
EMT1	It is pleasant/I like to use the application.
EMT2	The application allows the user to feel happy using it.

Engagement Dimension	
**Item**	**Description**
EGT1	The application arouses the interest in obtaining it.
EGT2	The application stimulates the desire to recommend it to others.
EGT3	The application stimulates the curiosity to know it more.

Innovative Dimension	
**Item**	**Description**
INO1	The application has innovative features (different ways to meet the user's need).

Social Dimension	
**Item**	**Description**
SOC1	The application lets you share information with others.
SOC2	The application allows being always updated (aware) about the contents it provides.
SOC3	The application is known and widely used by other people.

Physical Characteristics Dimension (Applicable for Mobile Applications)		
**Item**	**Description**	**Do these items apply to the evaluated app?**
PSC1	The application has good battery management (i.e., it does not consume a lot of battery).	( ) Yes<br>( ) No
PSC2	The application allows/enables the use of sensors to provide interaction in different ways: through GPS (location), accelerometer (movement), gyroscope (gestures) and voice recognition.	( ) Yes<br>( ) No

Learning and Ease of Use Dimension	
**Item**	**Description**
LEU1	The application interface is consistent (i.e., same interface items represent the same things).
LEU2	The application content (text, images, information, icons) are displayed in a visible and understandable way.
LEU3	The app's features do what they seem to do.
LEU4	The application is easy enough to perform the activities without difficulties.
LEU5	The application visibly provides tips or guides on how to use it.
LEU6	The application does not require much mental effort to remember how to use it.

Utility Dimension	
**Item**	**Description**
UTL1	The application assists in an important activity.

Control Dimension	
**Item**	**Description**
CTR1	The application allows controlling the interaction the way the user wants.

Feedback Dimension	
**Item**	**Description**
FCK1	The application provides information about the actions the user performs.
FCK2	Information about user actions is objective and understandable.

Dimension Efficiency	
**Item**	**Description**
EFF1	The application processes the information quickly.
EFF2	The application allows using shortcuts to perform some activities.

Value Added Dimension	
**Item**	**Description**
VLE1	The application generates value (has benefits that make the user prefer this application over the competitors).
VLE2	The application has/represents values that are important to the user.

Satisfaction Dimension	
**Item**	**Description**
STF1	The application meets user’s expectations.
STF2	The application fulfills what it is expected to do.

3 IHI Technique

Accessibility

1. **Perceivable:** the interface is presented in a way the user can always perceiver.

2. **Operable:** user interface components must be easy to use.

3. **Understandable:** the information presented must be easy to understand for all users.

4. **Robust:** the system must be robust enough for it to resist any unexpected solicitations.

Usability/Practicability

1. **Guidance:** means available to advise, orient, inform and guide the user throughout their interactions.

2. **Workload:** interface elements implicated in the reduction of the user's perceptual/cognitive load, and in the increase of dialogue efficiency.

3. **Explicit control:** system's ability to process the user's explicit actions, and the control they have over the processing of their actions.

4. **Adaptability**: system's capacity to behave contextually and accordingly to the user's needs/preferences.

5. **Error management:** means available to prevent or reduce errors and to recover from them when they do occur.

6. **Consistency:** interface design choices are maintained in similar contexts, but differ when the context is different.

7. **Significance of codes:** adequacy between a term/sign and its reference.

8. **Compatibility:** match between the user's and the task's characteristics, and the organization of the output, input and the dialogue for a given application.

Emotions & Motivation

1. **Control:** system's capacity to respect the degree of control the user likes having over it.

2. **Challenge:** system's ability to incite a challenging situation that the user enjoys be-ing confronted with.

3. **Independency:** system's capability to encourage the user to work independently without asking others for help.

4. **Fantasy:** the system evokes mental images of social/physical situations that are satisfying to the user.

5. **Confidence:** system's ability to arouse a required level of belief in being able to perform the task properly.

6. **Sensory Interest:** amount of curiosity aroused by the system's characteristics (interface, graphics, sounds, etc.).

7. **Cognitive interest:** curiosity aroused through the cognitive/epistemic characteristics of the task;

8. **Effort:** system's ability to inspire and maintain a certain level of effort needed for the user to perform the task.

9. **Satisfaction:** overall feeling of goal accomplishment procured by the system.

Persuasion

1. **Credibility:** ability of the interface to inspire confidence and make the user trust the veracity of its information.

2. **Privacy:** the system guarantees the protection of personal data, the preservation of personal integrity and the security of the interaction.

3. **Personalization:** the ability for a user to adapt the interface to their preferences.

4. **Attractiveness:** via the use of aesthetics, the system captures the user's attention in order to support the interaction and to create a positive emotion.

5. **Solicitation:** ability to induce a user action with minimal influence from the interface.

6. **Priming:** system's capacity to pilot the user's initiative in order to encourage them to execute the first engaging action(s).

7. **Commitment:** the system progressively increases the frequency and intensity of its requests in order to gradually involve the user more and more.

8. **Ascendency:** the system attains such a level of influence over the user that they be-come subject to a total level of involvement with the system.

4 Problem Table Form

The UX-Tips technique provides a form to report the problems that users encountered during the UX evaluation. We have compared the UX-Tips with IHI technique in the comparative study. However, IHI does not provide an artifact or a standardized way to report the problems encountered through its use. In this sense, we used the same UX-Tips form so that IHI users could report the problems found. Bellow, we present the UX-Tips form to report UX problems. In the first column, users enter the technique item code to which the problem is related. In the second column, the users describe the problems that they have encountered.

Table for identified issues

Technique Item Code	Problem Description (Describe the problem you encountered)

5 Activity Script

Below we present the activity script we used to guide the participants in the inspection process during the comparative study. We developed the activity script to allow the participants to evaluate the same interfaces. Thus, all participants followed the same flow of activities. We used the TripAdvisor script in the comparative study.

**App: TripAdvisor**	
**Activity**	**Description**
1	Search for attractions in any city you are interested in.
2	Check out the top 5 attractions of this city.
3	Choose one of the attractions to view its reviews.
4	Find a restaurant near the attraction you have chosen.
5	View restaurant's details such as: Average price, meals, and opening hours.

6 Feedback Questionnaire

We applied the Feedback questionnaire after all participants had completed the study. We made the questionnaire available online, with some additional questions to identify the participants, such as: (i) Name and (ii) E-mail.

Question	Description
1	Do you consider that the technique you used allowed you to evaluate the user experience? Describe the parts of the technique that you thought were helpful.
2	Do you consider that you were able to indicate the problems or difficulties of the application you used through the UX evaluation technique? Explain what could not be possible to indicate.
3	In your opinion, did you consider the technique easy to use? Were the item descriptions adequate/easy to understand?
4	Do you have any suggestions for improving the technique you used?

7 Personas

We present in this section the detailed information about the personas we used to create the activity script of the industry study. Due to the blind review policy, we omitted the name of both personas. Instead, we named the personas as persona 1 and persona 2. We put an "X" in cases where we could not provide much information, such as the name of the city where they live.

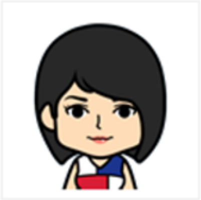

Name: X
Age: 40 years old

Demographics: Persona 1 is 40 years old and lives in the city of X. She has a high education. She is a teacher at a state school. Despite being patient, Persona 1 does not like to waste time.
Technology Experience: Persona 1 accesses the internet infrequently. They have no experience with computer or notebook use. She uses a smartphone with Android daily. She does not feel safe using apps that she does not know and also has afraid of making mistakes, but she can turn around. She uses a few applications in her daily life, including Facebook, because she likes to like and comment on the photos of her friends and family and she uses WhatsApp to communicate with friends and family group. Sometimes she visits local news sites.

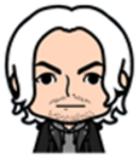

Name: X
Age: 60 years old

Demographics: Persona 2 is 60 years old and lives in municipality X. Has a high school education. He is self-employed and does not declare monthly income. He has vision difficulty due to his age.
Technology Experience: Persona 2 rarely accesses the internet. He has no experience with computers or notebooks. He uses his Android smartphone only when needed. Rarely uses applications. He only uses WhatsApp and, when he can not do something on his phone gets stressed and asks his child to help him.

8 Raw data

In this section, we provide two tables that contain the data of each participant regarding the comparative study. Each table contains all the data that we used to perform the quantitative analysis by technique. We classified the experience in both industry and UX evaluation as Low (L), Medium (M), and High (H).

Table S1. Data regarding UX-Tips participants

Group 01 – Subjects who used UX-Tips																	
**ID Subject**	**P01**	**P02**	**P03**	**P04**	**P05**	**P06**	**P07**	**P08**	**P09**	**P10**	**P11**	**P12**	**P13**	**P14**	**P28**	**P30**	**P35**
**Experience in Industry**	L	L	L	L	L	L	L	L	M	L	M	H	M	L	M	L	L
**Experience in UX evaluation**	M	M	M	H	H	M	H	M	M	H	H	H	H	M	H	H	H
**Total problems**	3	13	5	9	7	14	9	15	9	8	9	7	7	15	3	2	8
**False positives**	0	0	0	0	2	1	0	0	0	0	0	0	0	0	0	0	0
**Time (min)**	32	55	156	65	54	69	85	48	28	42	71	39	51	67	31	29	42
**Efficiency (defects/hour)**	5.6	13.9	1.9	8.3	7.8	12.2	6.4	18.8	19.3	11.4	7.6	10.8	8.2	13.4	5.81	4.14	11.4
**Effectiveness (%)**	3.0	13.0	5.0	9.0	7.0	14.0	9.0	15.0	9.0	8.0	9.0	7.0	7.0	15.0	2.16	1.44	5.76

**ID Subject**	**P36**	**P37**	**P38**	**P39**	**P40**	**P41**	**P42**	**P43**	**P44**	**P45**	**P46**	**P47**	**P48**	**P49**	**P50**	**P51**	**P53**
**Experience in Industry**	L	L	L	H	L	L	L	L	L	L	M	L	L	L	L	L	L
**Experience in UX evaluation**	H	M	M	H	M	M	M	M	M	M	M	H	M	M	H	M	M
**Total problems**	3	21	6	5	2	6	16	12	7	8	7	12	5	2	11	9	6
**False positives**	0	0	0	0	0	0	3	0	0	0	0	0	0	0	0	0	0
**Time (min)**	44	68	45	54	41	49	68	60	34	41	37	49	35	83	48	63	42
**Efficiency (defects/hour)**	4.09	18.5	8	5.56	2.93	7.35	14.1	12	12.3	11.7	11.3	14.6	8.57	2.17	13.7	8.57	8.57
**Effectiveness (%)**	2.16	15.1	4.32	3.6	1.44	4.32	11.5	8.63	5.04	5.76	5.04	8.63	3.6	2.16	7.91	6.47	4.32

Table S2. Data regarding IHI participants

Group 02 – Subjects who used IHI																	
**ID Subject**	**P15**	**P16**	**P17**	**P18**	**P19**	**P20**	**P21**	**P22**	**P23**	**P24**	**P25**	**P26**	**P27**	**P31**	**P32**	**P33**	**P34**
**Experience in Industry**	H	L	L	L	L	L	M	M	L	H	L	M	L	H	L	M	L
**Experience in UX evaluation**	H	H	M	M	M	H	M	H	H	H	H	M	M	H	H	H	M
**Total problems**	11	11	8	11	9	14	7	11	9	4	4	4	3	2	2	2	5
**False positives**	1	2	0	21	1	5	2	2	0	0	0	0	0	0	0	0	4
**Time (min)**	75	77	55	90	120	110	45	55	59	51	60	58	30	22	35	19	31
**Efficiency (defects/hour)**	8.80	8.57	8.73	7.33	4.5	7.64	9.33	12	9.15	4.71	4	4.14	6	5.45	3.43	6.32	9.68
**Effectiveness (%)**	7.91	7.91	5.76	7.91	6.47	10	5.04	7.91	6.47	2.88	2.88	2.88	2.16	1.44	1.44	1.44	3.6

**ID Subject**	**P54**	**P55**	**P56**	**P57**	**P58**	**P59**	**P60**	**P61**	**P62**	**P63**	**P64**	**P65**	**P66**	**P67**	**P68**	**P69**	**P70**
**Experience in Industry**	L	L	L	L	L	L	L	L	L	L	L	L	L	L	L	L	L
**Experience in UX evaluation**	M	M	M	M	M	M	M	M	M	M	H	M	M	M	H	M	M
**Total problems**	5	1	8	6	4	10	3	11	5	5	4	5	4	5	5	8	5
**False positives**	0	3	0	0	0	0	0	0	0	0	0	0	0	0	0	0	0
**Time (min)**	40	57	55	46	34	62	38	58	58	41	49	53	43	39	33	57	53
**Efficiency (defects/hour)**	7.5	1.05	8.73	7.83	7.06	9.68	4.74	11.3	5.17	7.32	4.9	5.66	5.58	7.69	9.09	8.42	5.66
**Effectiveness (%)**	3.6	0.72	5.76	4.32	2.88	7.19	2.16	7.91	3.6	3.6	2.88	3.6	2.88	3.6	3.6	5.76	3.6
